# Tauroursodeoxycholic acid prevents *Burkholderia pseudomallei*-induced endoplasmic reticulum stress and is protective during melioidosis in mice

**DOI:** 10.1186/s12866-021-02199-x

**Published:** 2021-05-04

**Authors:** Siqi Yuan, Yao Fang, Mengling Tang, Zhiqiang Hu, Chenglong Rao, Jiangao Chen, Yupei Xia, Meijuan Zhang, Jingmin Yan, Bin Tang, Xiaoyi He, Jianping Xie, Xuhu Mao, Qian Li

**Affiliations:** 1 Department of Clinical Microbiology and Immunology, College of Pharmacy and Medical Laboratory, Army Medical University (Third Military Medical University), Chongqing, 400038 China; 2 Department of Respiratory, General Hospital of Center Theater Command, Wuhan, 400070 China; 3 Institute of Modern Biopharmaceuticals, State Key Laboratory Breeding Base of Eco-Environment and Bio-Resource of the Three Gorges Area, Key Laboratory of Eco-environments in Three Gorges Reservoir Region, Ministry of Education, School of Life Sciences, Southwest University, Beibei, Chongqing, 400715 China; 4 Department of General Medicine, Southwest Hospital, Army Medical University (Third Military Medical University), Chongqing, 400038 China

**Keywords:** *Burkholderia pseudomallei*, Tauroursodeoxycholic acid, Endoplasmic reticulum stress, Apoptosis, Survival

## Abstract

**Background:**

*Burkholderia pseudomallei*, a facultative intracellular bacterium, is the aetiological agent of melioidosis that is responsible for up to 40% sepsis-related mortality in epidemic areas. However, no effective vaccine is available currently, and the drug resistance is also a major problem in the treatment of melioidosis. Therefore, finding new clinical treatment strategies in melioidosis is extremely urgent.

**Results:**

We demonstrated that tauroursodeoxycholic acid (TUDCA), a clinically available endoplasmic reticulum (ER) stress inhibitor, can promote *B. pseudomallei* clearance both in vivo and in vitro. In this study, we investigated the effects of TUDCA on the survival of melioidosis mice, and found that treatment with TUDCA significantly decreased intracellular survival of *B. pseudomallei*. Mechanistically, we found that *B. pseudomallei* induced apoptosis and activated IRE1 and PERK signaling ways of ER stress in RAW264.7 macrophages. TUDCA treatment could reduce *B. pseudomallei*-induced ER stress in vitro, and TUDCA is protective in vivo.

**Conclusion:**

Taken together, our study has demonstrated that *B. pseudomallei* infection results in ER stress-induced apoptosis, and TUDCA enhances the clearance of *B. pseudomallei* by inhibiting ER stress-induced apoptosis both in vivo and in vitro, suggesting that TUDCA could be used as a potentially alternative treatment for melioidosis.

**Supplementary Information:**

The online version contains supplementary material available at 10.1186/s12866-021-02199-x.

## Introduction

*Burkholderia pseudomallei*, a gram-negative bacillus, is the causative agent of a broad spectrum of clinical manifestations collectively known as melioidosis. *B. pseudomallei* is found mainly in soil and surface water in almost all tropical area in the world. The treatment of melioidosis has long been complicated owning to the broad-spectrum antibiotic resistance in *B. pseudomallei*, which further heightens concerns about possible emerging public health threat. Besides, *B. pseudomallei* has been classified as Tier 1 select agents by the US CDC considering its potential biohazard [1]. Therefore, it is urgent to develop new clinical therapeutic strategies in melioidosis. TUDCA is a hydrophilic bile acid that is normally produced by combination of taurine to ursodeoxycholic acid (UDCA) endogenously in humans in the liver [2]. Recent studies indicated that TUDCA can be used clinically in hepatobiliary diseases [3], and approved by the US Food and Drug Administration to be safely used as a drug for human intake [4]. In addition, TUDCA has also been reported to be effective in several other diseases, including vascular diseases, osteoarthritis, diabetes and neurodegenerative diseases [5–7]. Mechanistically, TUDCA reduces ER stress, effectively protects hepatocytes and restores glucose homeostasis in the pathogenesis of obesity, insulin resistance and diabetes mellitus and infectious diseases. Particularly, in the treatment of infectious diseases, TUDCA is implicated as a therapy for hepatitis B and C virus infection [8, 9], and also inhibits influenza A viral infection by disrupting viral M2 or IRE1 stress pathway [10, 11]. However, little is known about the effect of TUDCA in *B. pseudomallei* infection.

ER stress is an adaptive response to adjust ER functional capacity, which is initiated by the accumulation of misfolded and unfolded proteins in the ER [12]. To maintein protein folding homeostasis, the misfolded and unfolded proteins are binbed to the chaperone BiP, and then dissociated from three ER transmembrane proteins inositol requiring kinase 1 (IRE1), protein kinase R-like endoplasmic reticulum kinase (PERK) and activating transcription factor 6 (ATF6) [13]. IRE1 is an endonuclease that cleaves nucleotides from the X-box binding protein 1 (XBP1) mRNA, which affects the expression of downstream gene CHOP (C/EBP homologous protein, enhancer binding protein homologous protein, is also a transcription regulator) [13–16]. Then activated ATF6 results in its translocation to the Golgi, where site-specific proteases cleave it [17]. The activation of PERK results in translation attenuation. ER is a nutrient-rich organelle lacking of antimicrobial functions, some bacterial pathogens take advantage of its nature to ensure their intracellular survival and proliferation [13]. Smith JA et al. showed that counteracting the ER stress reduces *Brucella* intracellular growth [14]. Furthermore, IRE1α, identified by RNAi screening, is necessary for *Brucella* intracellular growth [18]. Besides, other study suggests the ER stress pathway plays an important role in the persistence of *Mycobacterium tuberculosis* (Mtb). This study indicates that eIF2α/CHOP pathway contributes to Mtb intracellular survival of in macrophages [19]. However, the relevance of the ER stress to *B. pseudomallei* replication in host cells remains unknown.

In this study, we evaluated the effects of TUDCA on the *B. pseudomallei* clearance in vivo and in vitro. We detected that the treatment of TUDCA increased the survival rate of melioidosis mice, and decreased the bacterial loads and inflammatory response of the lungs, spleen and liver tissues. Furthermore, we found that *B. pseudomallei* infection induced apoptosis and ER stress in RAW264.7 macrophages. And TUDCA treatment could inhibit ER stress after *B. pseudomallei* infection. Taken together, these results indicate that TUDCA promotes *B. pseudomallei* clearance and inhibits *B. pseudomallei*-induced ER stress, which may provide an alternative treatment for melioidosis.

## Materials and methods

### Cell lines and bacterial strains

The murine macrophage RAW264.7 cell line (Cat. TIB-71) was obtained from American Type Culture Collection. RAW264.7 cells were cultured in high glucose DMEM medium (Gibco, 11,965–092) containing 10% fetal bovine serum (FBS; Gibco, 10,100–147), and cultured at 37 °C and 5% CO_2_. In this study, the *B. pseudomallei* strain used is BPC006, a clinical isolate from a melioidosis patient in China [20]. *B. pseudomallei* was grown in Luria-Bertani (LB) with shaking at 37 °C overnight in a tightly conical flask (5 mL). The bacterial liquid was washed three times with phosphate-buffered saline (PBS) and centrifugation. The bacterial concentration was determined on optical density (OD) at 600 nm.

### Antibody and reagents

The primary antibodies used in this study as follow: the antibodies against β-actin (4970), Bip (3177), CHOP (5554), eIF2α (5324S), phospho-eIF2α (3597S), calreticulin (12238), caspase-3 (14220), and cleaved caspase-3 (9661) were purchased from Cell Signaling Technology. Alexa Fluor 555 (A-31570) and 488 (A-11029) secondary antibodies were purchased from Thermo Fisher Scientific using for immuno-fluorescence studies. The HRP-conjugated secondary antibody (7074) was purchased from Cell Signaling Technology. TUDCA was purchased from Solarbio (ST8580), and 4-phenylbutyric acid (4-PBA) was purchased from Sigma (P21005). The mouse polyclonal anti-*B. pseudomallei* antibody was generated in our lab [21].

### Animal experiments

For survival studies, a total of 12 C57BJ/6 mice aged 4–6 weeks were selected. Age and sex-matched animals were used in all experiments. In brief, C57BJ/6 mice were injected with PBS (control) or TUDCA (100 μg/g), and 1-day post-injection, mice were intranasally infected with about 5 × 10^3^ CFU of *B. pseudomallei*. After infection, TUDCA (100 μg/g) was injected every 2 days. Mice were monitored every day for 10 days during the experiment. The mice were observed daily and the number of mice died was recorded. After 10 days observation, the survivors were sacrificed. To assess the *B. pseudomallei* growth in organs, the lungs, spleen and liver tissues were homogenized separately with a tissue grinder in sterile PBS. The cell suspension was plated on LB plates and cultured at 37 °C for 72 h. Colony counts were obtained. Besides, the histological analysis of lungs, spleen, liver tissues were performed with H&E staining, and analyzed by a pathologist who was blinded as to groups. According to previously published criteria [22]: degree of inflammatory cell infiltration (normal, 0-dense inflammatory infiltrate, 3). The total histologic scores were obtained by summing each individual score. Experiments performed in twice showed consistent results. All *B. pseudomallei* infections experiments were approved by Institutional Biosafety Committee of Army Medical University, and performed in biosafety level 2+ (BSL-2+) laboratory spaces.

### Intracellular survival analysis of bacteria

RAW264.7 cells were infected with *B. pseudomallei* at an MOI of 10 for 2, 4, 6, 8 and 10 h. The extracellular bacteria were removed 1 h after infection, cells were washed with PBS three times, and 3 mL fresh culture medium containing 250 μg/mL of kanamycin was added. At the indicated time points, the infected cells were washed three times with PBS and lysed with 1 mL of 0.1% Triton X-100 (Sigma). The diluted lysates were plated on LB plates and the colonies were counted after 36 h. Colony counts were performed in triplicate.

### Annexin V-FITC/PI staining assay

The ratio of apoptosis cells was measured with an Annexin V-FITC/PI Detection Kit (BD Biosciences) according to the manufacturer’s instruction. Briefly, the collected cells were washed twice with cold PBS and then 1 × Binding Buffer was used to prepare 1 × 10^5^ cell/mL suspension. Each sample was supplemented with 5 μL Annexin V-FITC and 5 μL PI, respectively. Mix gently and place it in a dark place for 15 min at room temperature. The apoptosis was detected by flow cytometry (BD FACScan Flow cytometer, United States) within 1 h. And the data were analyzed with CellQuest software (BD Biosciences).

### Quantitative RT-PCR

Quantitative RT-PCR (qRT-PCR) assays for the mRNA of Bip and CHOP were performed by using the PrimeScript RT-PCR kit (Takara, RR037A) in CFX96 real time PCR machines (Bio-Rad) [23]. The reactions were performed using the following parameters: 95 °C for 1 min followed by 40 cycles of 95 °C for 5 s, 60 °C for 5 s and 72 °C for 20 s. The mRNA level of Actin was used as an endogenous control for data normalization. Relative expression was calculated using the comparative threshold cycle method. The relative primers were shown in Table 1.

**Table 1:** Primers used in this study

Primer	Sequence	Product size (bp)	Efficiency
Bip	F:5′-GTGTGTGAGACCAGAACCGT-3’	77	95.2%
	R:5′-GCAGTCAGGCAGGAGTCTTA-3’		
CHOP	F:5′- GCCAGAATAACAGCCGGAAC-3’	138	92.4%
	R:5′- ACCGTCTCCAAGGTGAAAGG-3’		
β-actin	F:5′-CTGAGCACACAGCTGGACTG-3’	71	102.4%
	R:5′-AAGCTGGTGGTACCTGATGC-3’		

In order to determine specificity of primers designed in the study, agarose gel electrophoresis and melting curve analyses were performed in the qRT-PCR experiment [24]. All the primer pairs amplified the expected size of single PCR product (Fig. S1), and the specificity of amplicon was confirmed by the presence of single peak during melt curve (Fig. S2). The slopes of the standard curves were used to calculate the correlation coefficient (R^2^) and PCR efficiency (Fig. S3). The linear R^2^ for all the primers ranged between 0.998 and 0.999. Further, PCR efficiencies of primers ranged from 92 to 103% (Table 1).

### Western blot analysis

The protein levels of targets were determined by western blotting as described by Tang et al. with minor modification [25]. The cells were washed three times with PBS and then lysed in RIPA lysis buffer (150 mM NaCl, 50 mM Tris HCL, 1% SDS, 0.5% Benzonase endonuclease (Merck Millipore), 0.5% sodium desoxicholate, 0.1% Nonidet P-40 and phosphatase and protease inhibitor cocktails (Roche) on ice for 20 min and then incubated at 100 °C for 10 min. After centrifugation at 12000 g at 4 °C for 10 min, the protein concentration was determined by BCA Protein Assay (Thermo Fisher Scientific). The lysates were subjected to standard SDS-PAGE. After electrophoresis, protein was transferred to a PVDF membrane. The membranes were blocked with 5% non-fat milk in Tris-buffered saline (Sigma, T5912, pH 7.4) containing 0.05% Tween 20 (Sigma, P1379). Membranes were incubated with indicated primary antibodies (1:1000 dilution of β-actin, Bip, eIF2α, phospho-eIF2α, caspase-3, and cleaved caspase-3; 1:500 dilution of CHOP) overnight at 4 °C, then with 1:8000 dilution of HRP-conjugated anti-rabbit antibodies for 2 h at room temperature. We used a commercial protein marker to identify the size of proteins. The protein of interest was visualized using the Supersignal® West Dura Duration substrate reagent (Thermo, 34,080).

### XBP1 mRNA splicing assay

To measure the degree of XBP1 mRNA splicing, the total cellular RNA was isolated. XBP1 was PCR amplified using AmpliTaq Gold™ 360 Master Mix (Thermo, 4,398,881), according to the manufacturer′s instructions. In order to amplify both the spliced and the unspliced variants of XBP1 cDNA, the primers were designed as follows: forward primer 5′-GCAAGTGGTGGATTTGGAAGA-3′, and reverse primer, 5′-GGAGGCTGGTAAGGAACTAGG-3′. For the analysis of PCR products, 10 μL of each reaction mixture was loaded on 2.5% agarose gel and subjected to electrophoresis to separate the products. The bands were visualized using GelTower Imager (Analytik Jena AG, Germany).

### Transmission electronic microscopy

The methods were described by Tang et al. with minor modification [25]. RAW264.7 cells were collected and fixed in 2% paraformaldehyde, 0.1% glutaraldehyde in 0.1 M sodium cacodylate for 2 h, postfixed with 1% O_S_O_4_ for 1.5 h, washed and stained for 1 h in 3% aqueous uranyl acetate. The samples were then washed again, dehydrated with a graded alcohol series, and embedded in Epon-Araldite resin (Canemco, 034). Ultrathin sections were cut on Reichert ultramicrotome (Reichert, United States), counterstained with 0.3% lead citrate. Finally, the samples were examined on a Philips EM420 electron microscope (Philips, United Kingdom).

### Immunofluorescence assay

RAW264.7 cells were pretreated with TUDCA (100 μM) or 4-PBA (250 μM) for 24 h, then infected with *B. pseudomallei* (MOI = 10) for 8 h. After infection, the cells were washed three times with PBS, fixed by incubation in 4% paraformaldehyde for 10 min, then permeated with 0.1% Triton X-100 for 10 min. Cells were washed three times with cold PBS and incubated with 1:100 dilution of anti-calreticulin antibody in 1% BSA overnight at 4 °C. Alexa Fluor 555 goat anti-rabbit IgG (1:1000) incubation for 1 h at room temperature. Subsequently, cells were incubated with 1:500 dilution of mouse polyclonal anti-*B. pseudomallei*, and incubated with 1:1000 dilution of Alexa Fluor 488 goat anti-mouse IgG for 1 h. Finally, the nuclear stain DAPI was applied for 10 min at room temperature. Washing three times with cold PBS followed each incubation. Cells were viewed by laser-scanning confocal microscopy (Zeiss, Germany). We acquired images in sequential scanning mode.

### MTT assay

RAW264.7 cells were seeded in 96-well culture plates according to the manufacturer’s instructions (Sigma, TOX1). After the treatment with 0, 20, 100, 200 and 500 μM of TUDCA for 24 h, the cells were incubated with 5 mg/mL MTT at 37 °C for 1 h. Then, the culture medium was removed, and the cells were dissolved in MTT solubilization solution (Sigma, TOX1). The absorbance was measured at 540 nm using a microplate reader (Bio-Rad).

### Statistical analysis

The results are expressed as mean ± SD from at least 3 separate experiments performed in triplicate. Student’s *t* test was used to analyzed the differences between two groups, but when multiple time points were compared, the ANOVA with a post-hoc test were used. SPSS 13.0 software was used for all statistical analysis. The differences were considered significant at *p* < 0.05. Statistically significant data are indicated by asterisks (*P < 0.05, **P < 0.01, ***P < 0.001).

## Results

### TUDCA facilitates *B. pseudomallei* clearance in vivo and in vitro

**Fig. 1:**
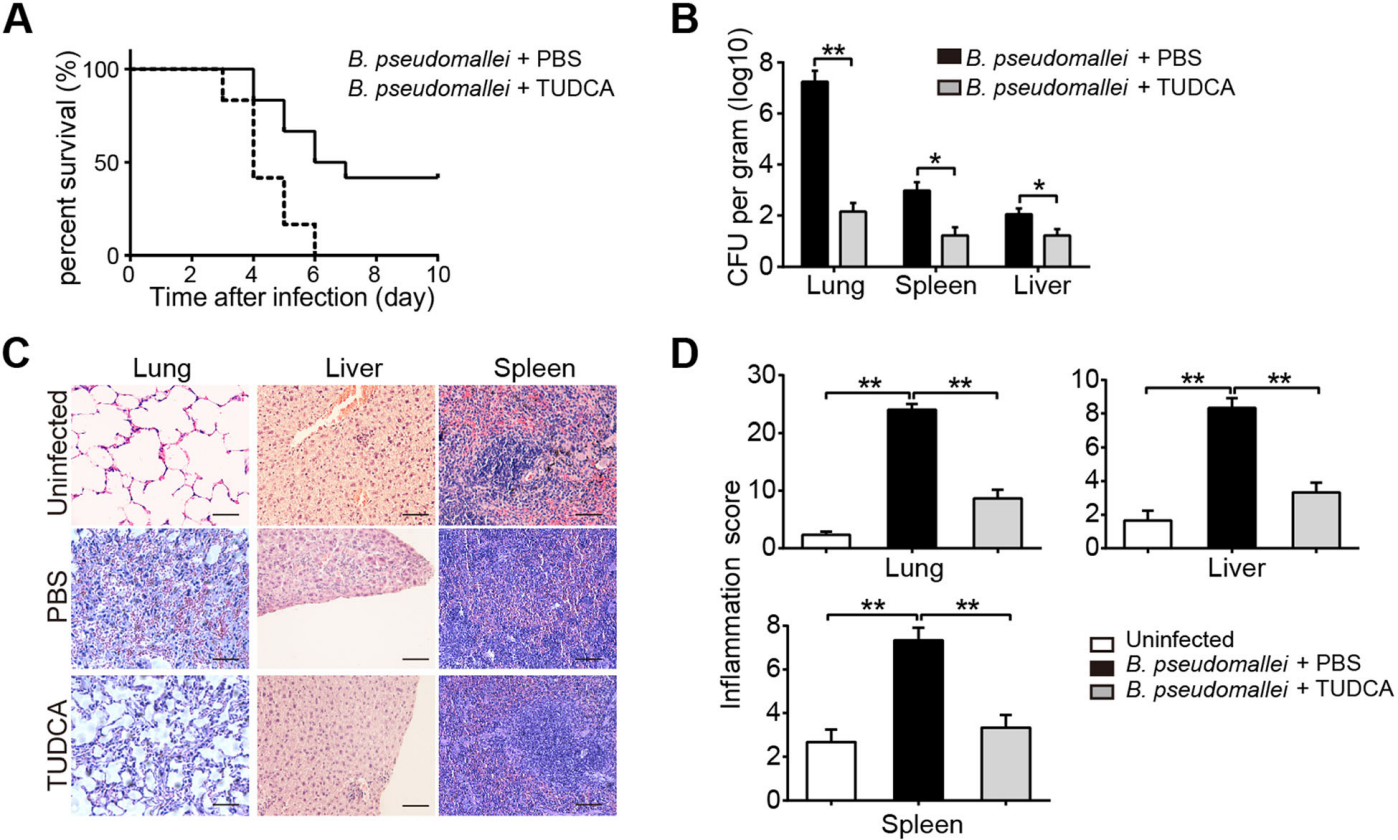
TUDCA treatment improves survival of mice during experimental melioidosis**.** Mice were injected with PBS or TUDCA (100 μg/g), and intranasally infected with about 5 × 10^3^ CFU of *B. pseudomallei*. Then TUDCA (100 μg/g) was injected every 2 days after infection. **a** Survival after intranasal infection with *B. pseudomallei* in the pretreatment of PBS or TUDCA. Mortality was assessed daily for 10 days. *n* = 6 per group. **b** The bacterial loads in lungs, liver and spleen of TUDCA or PBS-treated mice. **c** and **d** Representative H&E staining of lungs, liver, spleen and corresponding inflammation scores after 10 days infection. Scale bar is 50 μm. Data is shown as the mean ± SD of three independent experiments. *P < 0.05, **p < 0.01

**Fig. 2:**
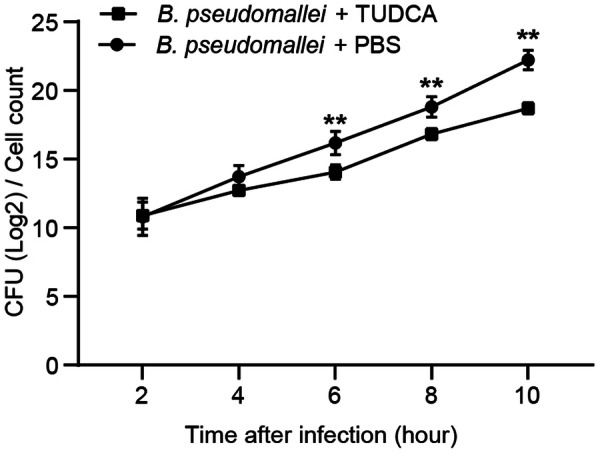
TUDCA reduces the intracellular survival of *B. pseudomallei* in RAW264.7 cells*.* RAW264.7 cells were treated with TUDCA (100 μM) for 24 h, and infected with *B. pseudomallei* (MOI = 10) for 2, 4, 6, 8 and 10 h. The intracellular bacteria were measured by CFU assay. The data are means ± SD of three independent experiments. **P < 0.01

To investigate the effects of TUDCA on the intracellular survival of *B. pseudomallei* in an in vivo mouse model, we firstly analyzed the survival curves of C57BJ/6 mice. All PBS treated mice in the survival experiment died by day 6 after inoculation, but mortality was delayed and reduced in TUDCA treated mice, of which 42% survived until the end of the 10 days observation period (Fig. 1a). Furthermore, as shown in Fig. 1b, bacterial loads in lungs, spleen and liver of TUDCA treated mice were significantly reduced when compared to the PBS control group following intranasally infected with *B. pseudomallei*. Next, to assess the inflammatory response of organs, histological analysis showed a stronger inflammation of lungs, spleen and liver in PBS treated mice, as reflected by more inflammatory cells infiltration; whereas TUDCA treatment markedly reduced these pathological changes and limited the inflammation, resulting in a decreased histological score (Fig. 1c and d). In addition, we also detected the role of TUDCA in *B. pseudomallei* infected RAW264.7 macrophages. As shown in Fig. 2, TUDCA treatment significantly reduced the intracellular survival of the *B. pseudomallei* in RAW264.7 cells. Taken together, these results suggest that TUDCA may play a protective role against melioidosis by facilitating *B. pseudomallei* eradication in vivo and in vitro.

### *B. pseudomallei* infection induces apoptosis in RAW264.7 macrophages

**Fig. 3:**
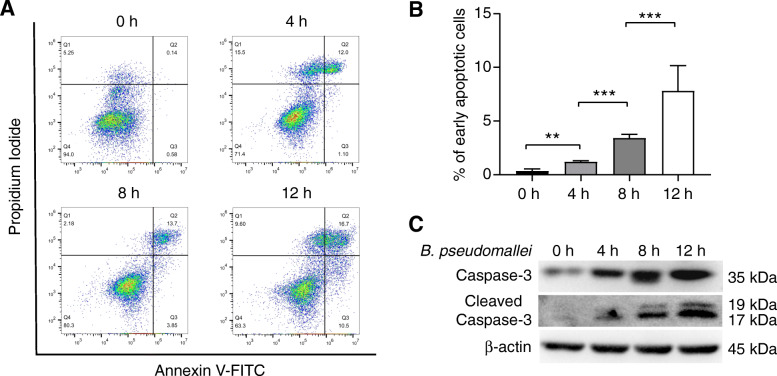
*B. pseudomallei* infection induces apoptosis of RAW264.7 cells. **a** RAW264.7 cells were infected with *B. pseudomallei* (MOI = 10) for 4, 8 and 12 h, and then stained with Annexin V-FITC/PI and analyzed by flow cytometry. **b** The percentage of early apoptotic cells relative to the total number of cells was counted. **c** Measurement of the caspase-3 and cleaved caspase-3 in RAW264.7 cells subjected to the indicated treatments by western blot analysis. The full-length blots are presented in Supplementary Fig. S4. The data obtained are at least 3 independent experiments. The ANOVA with a post-hoc test was used to analyze multiple time points. **P < 0.01, ***P < 0.001

Previous studies have shown that *B. pseudomallei* infection induces the expression of apoptosis-related genes in mouse macrophages [26]. Therefore, we speculated that *B. pseudomallei* infection may undergo apoptosis in RAW264.7 cells. Firstly, we analyzed apoptosis of *B. pseudomallei* infected RAW264.7 cells by flow cytometry and western blot. The results of flow cytometry indicated that the proportion of early apoptotic cells increased in a time-dependent manner after *B. pseudomallei* infection (Fig. 3a and b). Meanwhile, caspase-3 is one of the effector caspases, a family of cysteine proteases, which plays a common role of death effector molecules in various forms of apoptosis. As shown in Fig. 3c, the western blot result indicated that *B. pseudomallei* infection induced the expression of caspase-3 and its active form cleaved caspase-3. Taken together, these data indicate that *B. pseudomallei* infection induces apoptosis in RAW264.7 cells.

### *B. pseudomallei* induces ER stress in RAW264.7 macrophages

**Fig. 4:**
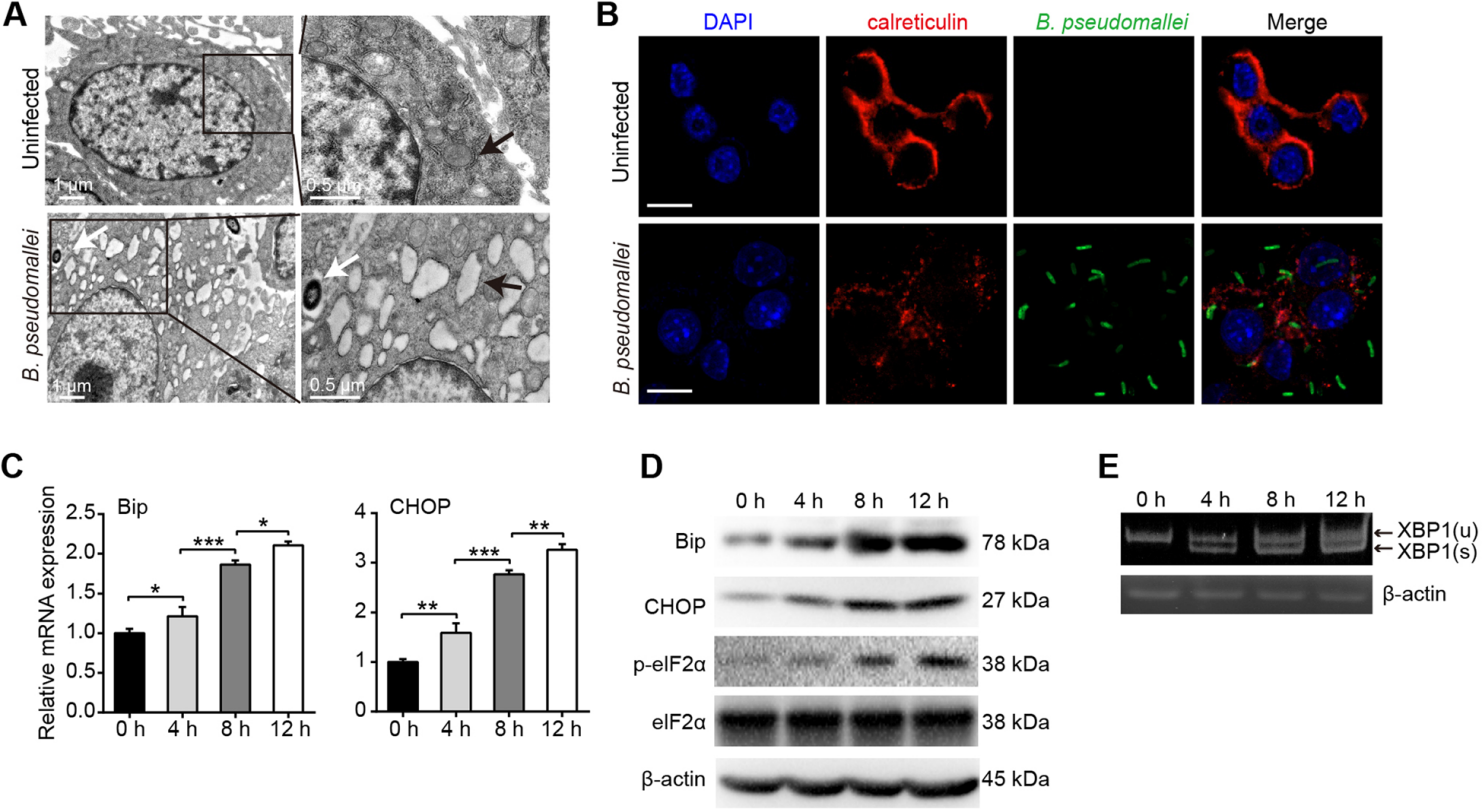
*B. pseudomallei* induces ER stress in RAW264.7 cells. **a** Representative TEM images of RAW264.7 cells after uninfected or infected with *B. pseudomallei* (MOI = 10) for 8 h. White arrowheads indicate the *B. pseudomallei*, and black arrowheads indicate the ER. **b** RAW264.7 cells were uninfected or infected with *B. pseudomallei* (MOI = 10) for 8 h. Cells were stained with anti-calreticulin antibody (red), anti-*B. pseudomallei* antibody (green) or DAPI (blue). Scale bar is 10 μm. **c** The mRNA expression of Bip, CHOP, in RAW264.7 cells infected with *B. pseudomallei* (MOI = 10) for 4, 8 and 12 h. The ANOVA with a post-hoc test was used to analyze multiple time points. **d** Immunoblot analysis of Bip, CHOP, elF2α and p-elF2α levels after *B. pseudomallei* infection (MOI = 10) for 4, 8 and 12 h. **e** Cells were infected with 10 MOI for 4, 8 and 12 h, and processed for RNA. XBP1 spliced and unspliced mRNA species were resolved by 2.5% agarose gel. The full-length blots or gels are presented in Supplementary Fig. S4 and S5. Results were measured at least 3 times. *P < 0.05, **P < 0.01, ***P < 0.001

The accumulation of unfolded proteins in the ER is toxic to cells. To determine whether *B. pseudomallei* infection could induce ER stress response in RAW264.7 cells, we firstly examined the morphologic alterations of ER structure. The TEM observations showed that ER enlargement in the *B. pseudomallei* infected cells (Fig. 4a). Meanwhile, as shown in Fig. 4b, fragmentation and condensation of ER was visualized with anti-calreticulin antibody after *B. pseudomallei* infection. Taken together, these results suggest that the shape-structure of ER is changed in responsible to *B. pseudomallei* infection in RAW264.7 cells.

Given that ER stress activation involves three major signaling pathways, stemming from activation of PERK and IRE1, we detected the mRNA and protein expression levels of the chaperone Bip and downstream target CHOP in *B. pseudomallei* infected RAW264.7 cells at 4, 8, 12 h, respectively. As shown in Fig. 4c, the results showed that the mRNA expression levels of Bip and CHOP were consistently upregulated. And the protein expressions of Bip, CHOP and p-eIF2α were increased in a time-dependent manner (Fig. 4d). Upon activation of IRE1, its endonuclease activity activated. The transcription factor XBP1 is spliced. Thus, we detected XBP1 spliced and unspliced mRNA species by 2.5% agarose gel. As shown in Fig. 4e, XBP1 mRNA was spliced after *B. pseudomallei* infection. Collectively, these findings indicate that *B. pseudomallei* infection could induce ER stress in RAW264.7 cells.

### TUDCA attenuates *B. pseudomallei*-induced ER stress in RAW264.7 cells

**Fig. 5:**
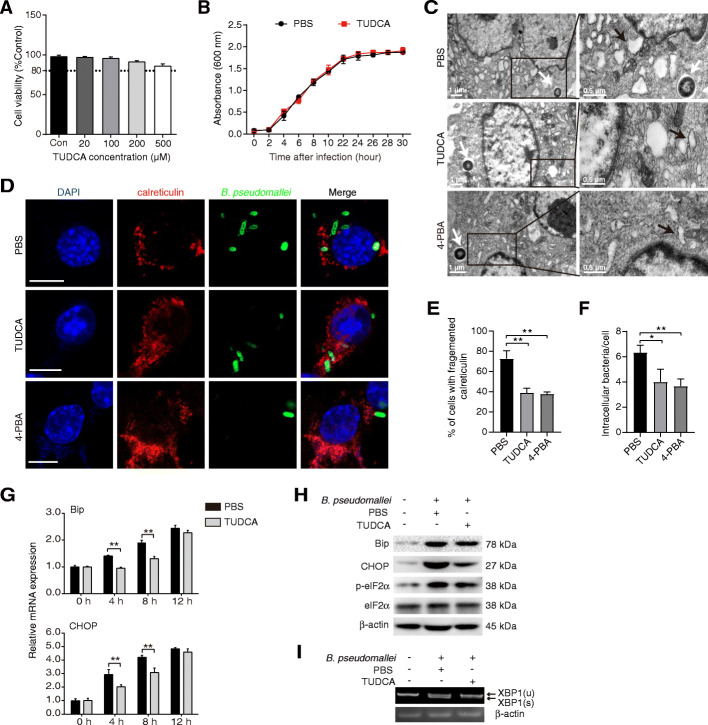
TUDCA inhibits ER stress induced by *B. pseudomallei*. **a** Effect of TUDCA on the cell viability. RAW264.7 cells were treated with TUDCA (0, 20, 100, 200, and 500 μM) for 24 h. **b** The effect of TUDCA on *B. pseudomallei* culture growth. *B. pseudomallei* were treated with 100 μM TUDCA for different time points. At respective time points optical density (OD) was measured at 600 nm. **c** Representative TEM images of ER in RAW264.7 cells. Cells were treated with TUDCA (100 μM), 4-PBA (250 μM) or PBS for 24 h, and infected with *B. pseudomallei* (MOI = 10) for 8 h. White arrowheads indicate the *B. pseudomallei*, and black arrowheads indicate the ER. **d** Immunofluorescence assay of calreticulin in RAW264.7 cells. Cells were treated as described above and stained with anti-calreticulin antibody (red), anti-*B. pseudomallei* antibody (green) or DAPI (blue). Scale bar is 10 μm. **e** Percentage of cells with fragmented calreticulin signal area, and the area of calreticulin as a function of the total cell area, were calculated in TUDCA, 4-PBA and PBS-treated cells (*n* > 50 cells per experiment). **f** The average of intracellular *B. pseudomallei* in each cell were counted. **g** The mRNA expression of Bip, CHOP in RAW264.7 cells infected with *B. pseudomallei* (MOI = 10) for 8 h. **h** Immunoblot analysis of Bip, CHOP, elF2α and p-elF2α levels after *B. pseudomallei* infection (MOI = 10) for 8 h. **i** Cells were infected with 10 MOI for 8 h, 2.5% agarose gel image shows unspliced and spliced (XBP1(u) and XBP1(s)) mRNA species. The full-length blots or gels are presented in Supplementary Fig. S4 and S5. Results are representative of 3 independent experiments. *P < 0.05, **P < 0.01

We assessed TUDCA cytotoxicity using the MTT assay in RAW264.7 cells, and found that TUDCA alone did not lead to cell death at concentrations between 0 and 500 μM (Fig. 5a). In addition, we did not observe a direct effect of TUDCA on *B. pseudomallei* growth (Fig. 5b). TUDCA is known to inhibit the ER stress by reducing the load of misfolded proteins in vitro. To assess whether TUDCA inhibits the ER stress induced by *B. pseudomallei*, the ER stress inhibitor 4-PBA was used as a positive control. As shown in Fig. 5c, the treatment of TUDCA and 4-PBA attenuated the ER expansion. Meanwhile, TUDCA treatment ameliorated the ER condensation and fragment, and similar effects were obtained in the presence of 4-PBA (Fig. 5d). To quantify the altered phenotype, the percentage of cells with fragmented calreticulin was calculated. Calreticulin was found fragmented to the cell periphery in 39% of TUDCA-treated and 37% of 4-PBA-treated cells, against 72% of PBS-treated cells (Fig. 5e). In addition, quantification of intracellular bacteria by immunofluorescence staining indicated that the TUDCA treatment reduced the bacterial load, which were similar to 4-PBA treatment (Fig. 5f).

Furthermore, the mRNA and protein expression levels of ER stress markers were evaluated. The mRNA expression levels of Bip and CHOP, were reduced subsequent to the TUDCA treatment (Fig. 5g). Meanwhile, the protein level of Bip, CHOP and p-eIF2α were reduced after TUDCA treatment (Fig. 5h). As shown in Fig. 5i, TUDCA treatment inhibited the splicing level of XBP1 induced by *B. pseudomallei*. Our results suggest that TUDCA treatment suppresses the ER stress induced by *B. pseudomallei* in RAW264.7 cells.

## Discussion

The awareness and global burden of melioidosis has long been underestimated, and difficulties in clinical recognition and laboratory diagnosis often lead to delays in treatment. Furthermore, considering no effective vaccine available and antimicrobial resistance for treating melioidosis currently, effective alternative drugs and new therapies are increasingly needed [1]. In this study, we have demonstrated that TUDCA is effective to control *B. pseudomallei* infection in mice. Meanwhile, in cultured cells, *B. pseudomallei* infection results in ER stress-induced apoptosis, and TUDCA enhances the clearance of *B. pseudomallei* by inhibiting ER stress. These results provide useful information for developing potential therapeutic strategies against *B. pseudomallei* infection.

Recent studies have revealed that bacterial pathogens have evolved multiple strategies to subvert host immune clearance responses by avoiding killing, including the proinflammatory response and manipulation of vesicular trafficking [27]. ER stress is a cytoprotective response to maintain the cellular homeostasis, and has been implied to participate in the modulation of innate immune signaling pathways and host defenses against invading microorganisms. Recent evidence shows these bacteria take advantage of the ER environment to became a safe niche for its intracellular survival, such as *Legionella*, *Brucella*, *Chlamydia* and *Simkania*. CR Roy et al. have reported that *Legionella*-containing vacuoles (LCVs) fuse with ER membranes through the endocytic pathway and disturb maturation to permit its intracellular replication, once upon phagocytosis by macrophages [28–30]. Similarly, J Celli et al. found that *Brucella* residing within a vacuole (*Brucella*-containing vacuole, BCV) traffics along the endocytic pathway and obtains the maturation properties of phagolysosome to form the ER-derived vacuoles, which supports bacterial proliferation [31, 32]. However, accumulating studies support the view that bacterial infections trigger ER stress and can be sensed via the unfolded protein response (UPR) [13]. Interestingly, so far, it is still not clear whether the induced-ER stress is beneficial to the intracellular survival of pathogens or the host defenses against bacterial infection.

Several studies have demonstrated that bacterial pathogens infection could induce ER stress and trigger UPR the for their own benefits. A previous study has reported that the virulence factor of *Helicobacter Pylori*, vacuolating cytotoxin A (VacA), is involved in the activation of ER stress by regulating PERK and eIF2α [33]. And *Brucella* induces ER stress by inducing three major signaling pathways of the UPR, which is correlated with the microtubule modulating protein TcpB [14]. Additionally, *Listeria monocytogenes* also induces the UPR and activates ER stress-specific apoptosis, via secretion of Listeriolysin O (LLO), by breaking intracellular Ca^2+^ homeostasis [34, 35]. These above results have indicated that bacterial infections induce the ER stress to promote their intracellular growth, but the underlying mechanisms remain to be further addressed.

Currently, the evidence has implied that the cellular downstream responses of ER stress are of high complexity, including production of reactive oxygen species, induction of autophagy, initiation of apoptosis, and stimulation of innate immune responses [13, 36, 37]. The protein TcpB of *Brucella melitensis* has been reported to induce ER stress, but also interact with the TLR adapter MyD88-adapter-like (MAL) to inhibit the activation of NF-κB signaling pathway [38, 39]. In addition, reticulophagy (ER-phagy, a selective autophagy), as one of the ER stress-induced downstream responses, plays a dual role in the regulation of intracellular survival of bacteria. Under strong stress conditions, the failure to protect cells by ER-phagy eventually leads to apoptotic cell death, of which the effect on bacterial replication seems to be inconclusive. *Shigella flexneri*, *Salmonella typhimurium*, and *Yersinia enterocolitica* have been proved to be able to promote apoptosis. Particularly, *Salmonella* and *Shigella* directly activates the pro-apoptotic signaling pathway to initiate apoptosis; while *Yersinia* kills host cells by inhibiting survival pathways and activating apoptotic signaling pathways [40]. Previous studies indicate that inducing apoptosis in macrophages may protect bacteria from being swallowed and promote bacterial survival, which enabling bacteria to reach submucosa, even blood and other organs [41]. Besides, the evidence indicates that inducing apoptosis in macrophages rather than necrosis will not attract more inflammatory cells infiltrations [41], implying that inhibition of apoptosis may not be conducive to the survival of bacteria. Consistent with the previous studies [26, 42, 43], our findings also confirmed that *B. pseudomallei* induced apoptosis in RAW264.7 cells in a time-dependent manner. In general, the detailed mechanisms of the interaction between bacterial infection and the host ER stress are complicated and need to be investigated in the future. Therefore, future work is likely to reveal which virulence factors are used to manipulate host ER stress, and which new strategies are utilized to regulate the ER stress-mediated immune inflammatory response.

In order to understand the relationship between *B. pseudomallei* and ER stress, we observed that *B. pseudomallei* infection activates ER stress by the three main signaling ways in RAW264.7 macrophages. Considering the crucial role of ER stress in infectious diseases, TUDCA, an ER stress inhibitor, implies the potential role in clinical treatment of *B. pseudomallei* infection. Indeed, JA Smith et al. have founded that the treatment of TUDCA decreases the intracellular replication of *B. melitensis* [14]. According to our observations, inhibiting the ER stress by TUDCA or 4-PBA treatment promotes the intracellular clearance of *B. pseudomallei*. Mechanistically, recent studies have reported that TUDCA mediates several diseases by suppressing ER stress, inhibiting cell apoptosis or modulating other molecular mechanisms, including influenza A viral infection, chronic viral hepatitis C, osteoarthritis, kidney disease and diabetes [8, 11, 44–46]. Of note, TUDCA as a clinically available ER stress inhibitor, is implied the potential role in clinical treatment of *B. pseudomallei* infection, which currently requires long-term intravenous and oral antibiotic courses. TUDCA improves the ability of cells to deal with unfolded proteins, reduces cell apoptosis and exerts the protective effect on cells. But the possible mechanism by which TUDCA is effective to *B. pseudomallei* infection is currently unknown.

In conclusion, this study highlights that *B. pseudomallei* infection induces apoptosis and ER stress in macrophages. TUDCA diminishes *B. pseudomallei*-induced ER stress and promotes the intracellular clearance of *B. pseudomallei* in vitro, and plays a protective role for host to control infection in vivo. Therefore, these insights provide a potential therapeutic strategy to treat melioidosis.

## Supplementary Information

**Additional file 1: Figure S1.** The confirmation of expected amplicon size of the primer pairs. The primer pairs were tested before qRT-PCR, using cDNA by standard PCR reaction with SimpliAmp Thermal Cycler (Thermofisher). The amplicons sizes were verified in 2% agarose gel electrophoresis. **Figure S2.** Melt curves of the Bip, CHOP and actin genes. **Figure S3.** Amplification efficiencies of the qRT-PCR primers designed in the study. **Figure S4.** The primary images for the cropped blots in Figs. 3c, 4d and 5h. **Figure S5.** The primary images for the cropped gels in Figs. 4e and 5i. **Figure S6.** The primary confocal data for the cropped images in Figs. 4b and 5d.

## Data Availability

All data generated or analyzed during this study are included in this published article and its supplementary information files.

## References

[1] Wiersinga WJ, Virk HS, Torres AG, Currie BJ, Peacock SJ, Dance DAB, Limmathurotsakul D (2018). Melioidosis. Nature Rev Dis Primers.

[2] Elia AE, Lalli S, Monsurro MR, Sagnelli A, Taiello AC, Reggiori B, La Bella V, Tedeschi G, Albanese A (2016). Tauroursodeoxycholic acid in the treatment of patients with amyotrophic lateral sclerosis. Eur J Neurol.

[3] Heubi JE, Wiechmann DA, Creutzinger V, Setchell KD, Squires R, Couser R, Rhodes P (2002). Tauroursodeoxycholic acid (TUDCA) in the prevention of total parenteral nutrition-associated liver disease. J Pediatr.

[4] Yoon YM, Lee JH, Yun SP, Han YS, Yun CW, Lee HJ, Noh H, Lee SJ, Han HJ, Lee SH (2016). Tauroursodeoxycholic acid reduces ER stress by regulating of Akt-dependent cellular prion protein. Sci Rep.

[5] Ozcan U, Yilmaz E, Ozcan L, Furuhashi M, Vaillancourt E, Smith RO, Gorgun CZ, Hotamisligil GS (2006). Chemical chaperones reduce ER stress and restore glucose homeostasis in a mouse model of type 2 diabetes. Science.

[6] De Miguel C, Sedaka R, Kasztan M, Lever JM, Sonnenberger M, Abad A, Jin C, Carmines PK, Pollock DM, Pollock JS (2019). Tauroursodeoxycholic acid (TUDCA) abolishes chronic high salt-induced renal injury and inflammation. Acta Physiol.

[7] Rodrigues CM, Sola S, Nan Z, Castro RE, Ribeiro PS, Low WC, Steer CJ (2003). Tauroursodeoxycholic acid reduces apoptosis and protects against neurological injury after acute hemorrhagic stroke in rats. Proc Natl Acad Sci U S A.

[8] Picciotto A, Savarino V, Bardellini E, Borzone S, Pireddu M, Sinelli N, Celle G (1994). Effect of therapy with combined interferon and tauroursodeoxycholic acid in chronic hepatitis C: biochemical and virologic evaluation. Curr Ther Res-Clin Exp.

[9] Yan H, Peng B, Liu Y, Xu G, He W, Ren B, Jing Z, Sui J, Li W (2014). Viral entry of hepatitis B and D viruses and bile salts transportation share common molecular determinants on sodium taurocholate cotransporting polypeptide. J Virol.

[10] Hassan IH, Zhang MS, Powers LS, Shao JQ, Baltrusaitis J, Rutkowski DT, Legge K, Monick MM (2012). Influenza a viral replication is blocked by inhibition of the inositol-requiring enzyme 1 (IRE1) stress pathway. J Biol Chem.

[11] Li N, Zhang Y, Wu S, Xu R, Li Z, Zhu J, Wang H, Li X, Tian M, Lu H, Jin N, Jiang C (2019). Tauroursodeoxycholic acid (TUDCA) inhibits influenza a viral infection by disrupting viral proton channel M2. Sci Bull.

[12] Chovatiya R, Medzhitov R (2014). Stress, inflammation, and defense of homeostasis. Mol Cell.

[13] Celli J, Tsolis RM (2015). Bacteria, the endoplasmic reticulum and the unfolded protein response: friends or foes?. Nat Rev Microbiol.

[14] Smith JA, Khan M, Magnani DD, Harms JS, Durward M, Radhakrishnan GK, Liu YP, Splitter GA (2013). Brucella induces an unfolded protein response via TcpB that supports intracellular replication in macrophages. PLoS Pathog.

[15] Gething MJ (1999). Role and regulation of the ER chaperone BiP. Semin Cell Dev Biol.

[16] Lee AS (2005). The ER chaperone and signaling regulator GRP78/BiP as a monitor of endoplasmic reticulum stress. Methods.

[17] Hillary RF, FitzGerald U (2018). A lifetime of stress: ATF6 in development and homeostasis. J Biomed Sci.

[18] Qin QM, Pei J, Ancona V, Shaw BD, Ficht TA, de Figueiredo P (2008). RNAi screen of endoplasmic reticulum-associated host factors reveals a role for IRE1alpha in supporting Brucella replication. PLoS Pathog.

[19] Lim YJ, Choi JA, Choi HH, Cho SN, Kim HJ, Jo EK, Park JK, Song CH (2011). Endoplasmic reticulum stress pathway-mediated apoptosis in macrophages contributes to the survival of mycobacterium tuberculosis. PLoS One.

[20] Fang Y, Huang Y, Li Q, Chen H, Yao Z, Pan J, Gu J, Tang B, Wang HG, Yu B, Tong YG, Zou QM, Mao XH (2012). First genome sequence of a Burkholderia pseudomallei isolate in China, strain BPC006, obtained from a melioidosis patient in Hainan. J Bacteriol.

[21] Hu ZQ, Rao CL, Tang ML, Zhang Y, Lu XX, Chen JG, Mao C, Deng L, Li Q, Mao XH (2019). Rab32 GTPase, as a direct target of miR-30b/c, controls the intracellular survival of Burkholderia pseudomallei by regulating phagosome maturation. PLoS Pathog.

[22] Kim JJ, Shajib MS, Manocha MM, Khan WI. Investigating intestinal inflammation in DSS-induced model of IBD. J Vis Exp. 2012;2(60):e3678. 10.3791/3678. 10.3791/3678 PMC3369627 22331082

[23] Radonic A, Thulke S, Mackay IM, Landt O, Siegert W, Nitsche A (2004). Guideline to reference gene selection for quantitative real-time PCR. Biochem Biophys Res Commun.

[24] Chandna R, Augustine R, Bisht NC (2012). Evaluation of candidate reference genes for gene expression normalization in Brassica juncea using real time quantitative RT-PCR. PLoS One.

[25] Tang B, Li Q, Zhao XH, Wang HG, Li N, Fang Y, Wang K, Jia YP, Zhu P, Gu J, Li JX, Jiao YJ, Tong WD, Wang M, Zou QM, Zhu FC, Mao XH (2015). Shiga toxins induce autophagic cell death in intestinal epithelial cells via the endoplasmic reticulum stress pathway. Autophagy.

[26] Hseu YC, Sung JC, Shieh BS, Chen SC (2014). Burkholderia pseudomallei infection induces the expression of apoptosis-related genes and proteins in mouse macrophages. J Microbiol Immunol Infect.

[27] Baxt LA, Garza-Mayers AC, Goldberg MB (2013). Bacterial subversion of host innate immune pathways. Science.

[28] Tilney LG, Harb OS, Connelly PS, Robinson CG, Roy CR (2001). How the parasitic bacterium Legionella pneumophila modifies its phagosome and transforms it into rough ER: implications for conversion of plasma membrane to the ER membrane. J Cell Sci.

[29] Robinson CG, Roy CR (2006). Attachment and fusion of endoplasmic reticulum with vacuoles containing Legionella pneumophila. Cell Microbiol.

[30] Kagan JC, Roy CR (2002). Legionella phagosomes intercept vesicular traffic from endoplasmic reticulum exit sites. Nat Cell Biol.

[31] Starr T, Child R, Wehrly TD, Hansen B, Hwang S, Lopez-Otin C, Virgin HW, Celli J (2012). Selective subversion of autophagy complexes facilitates completion of the Brucella intracellular cycle. Cell Host Microbe.

[32] Starr T, Ng TW, Wehrly TD, Knodler LA, Celli J (2008). Brucella intracellular replication requires trafficking through the late endosomal/lysosomal compartment. Traffic.

[33] Akazawa Y, Isomoto H, Matsushima K, Kanda T, Minami H, Yamaghchi N, Taura N, Shiozawa K, Ohnita K, Takeshima F, Nakano M, Moss J, Hirayama T, Nakao K (2013). Endoplasmic reticulum stress contributes to helicobacter pylori VacA-induced apoptosis. PLoS One.

[34] Pillich H, Loose M, Zimmer KP, Chakraborty T (2012). Activation of the unfolded protein response by listeria monocytogenes. Cell Microbiol.

[35] Gekara NO, Groebe L, Viegas N, Weiss S (2008). Listeria monocytogenes desensitizes immune cells to subsequent Ca2+ signaling via listeriolysin O-induced depletion of intracellular Ca2+ stores. Infect Immun.

[36] Ding WX, Ni HM, Gao W, Hou YF, Melan MA, Chen X, Stolz DB, Shao ZM, Yin XM (2007). Differential effects of endoplasmic reticulum stress-induced autophagy on cell survival. J Biol Chem.

[37] Urano F, Wang X, Bertolotti A, Zhang Y, Chung P, Harding HP, Ron D (2000). Coupling of stress in the ER to activation of JNK protein kinases by transmembrane protein kinase IRE1. Science.

[38] Snyder GA, Deredge D, Waldhuber A, Fresquez T, Wilkins DZ, Smith PT, Durr S, Cirl C, Jiang J, Jennings W, Luchetti T, Snyder N, Sundberg EJ, Wintrode P, Miethke T, Xiao TS (2014). Crystal structures of the toll/Interleukin-1 receptor (TIR) domains from the Brucella protein TcpB and host adaptor TIRAP reveal mechanisms of molecular mimicry. J Biol Chem.

[39] Sengupta D, Koblansky A, Gaines J, Brown T, West AP, Zhang D, Nishikawa T, Park SG, Roop RM, Ghosh S (2010). Subversion of innate immune responses by Brucella through the targeted degradation of the TLR signaling adapter, MAL. J Immunol.

[40] Zhou ZJ, Sun L (2016). Edwardsiella tarda-induced inhibition of apoptosis: a strategy for intracellular survival. Front Cell Infect Microbiol.

[41] Grassme H, Jendrossek V, Gulbins E (2001). Molecular mechanisms of bacteria induced apoptosis. Apoptosis.

[42] Sun GW, Lu J, Pervaiz S, Cao WP, Gan YH (2005). Caspase-1 dependent macrophage death induced by Burkholderia pseudomallei. Cell Microbiol.

[43] Bast A, Krause K, Schmidt IH, Pudla M, Brakopp S, Hopf V, Breitbach K, Steinmetz I (2014). Caspase-1-dependent and -independent cell death pathways in Burkholderia pseudomallei infection of macrophages. PLoS Pathog.

[44] Li P, Fu D, Sheng Q, Yu S, Bao X, Lv Z (2019). TUDCA attenuates intestinal injury and inhibits endoplasmic reticulum stress-mediated intestinal cell apoptosis in necrotizing enterocolitis. Int Immunopharmacol.

[45] Arai Y, Choi B, Kim BJ, Rim W, Park S, Park H, Ahn J, Lee SH (2019). Tauroursodeoxycholic acid (TUDCA) counters osteoarthritis by regulating intracellular cholesterol levels and membrane fluidity of degenerated chondrocytes. Biomaterials science.

[46] Zhou Q, Wang D, Xu J, Chi B (2016). Effect of Tauroursodeoxycholic acid and 4-Phenylbutyric acid on metabolism of copper and zinc in type 1 diabetic mice model. Biol Trace Elem Res.

