# Supplementary material for: Tauroursodeoxycholic acid prevents Burkholderia pseudomallei-induced endoplasmic reticulum stress and is protective during melioidosis in mice
Source: BMC Microbiol. 2021 May 4;21:137. doi: 10.1186/s12866-021-02199-x (PMC8094575; doi:10.1186/s12866-021-02199-x)
Supplement: Supplementary file 1 — Additional file 1: Figure S1. The confirmation of expected amplicon size of the primer pairs. The primer pairs were tested before qRT-PCR, using cDNA by standard PCR reaction with SimpliAmp Thermal Cycler (Thermofisher). The amplicons sizes were verified in 2% agarose gel electrophoresis. Figure S2. Melt curves of the Bip, CHOP and actin genes. Figure S3. Amplification efficiencies of the qRT-PCR primers designed in the study. Figure S4. The primary images for the cropped blots in Figs. 3c, 4d and 5h. Figure S5. The primary images for the cropped gels in Figs. 4e and 5i. Figure S6. The primary confocal data for the cropped images in Figs. 4b and 5d. [file 12866_2021_2199_MOESM1_ESM.docx]

**Tauroursodeoxycholic acid prevents *Burkholderia pseudomallei*-induced endoplasmic reticulum stress and is protective during melioidosis in mice**

Siqi Yuan ^1,3†^, Yao Fang ^2†^, Mengling Tang ^1,3^, Zhiqiang Hu ^1^, Chenglong Rao ^1^, Jiangao Chen ^4^, Yupei Xia ^1^, Meijuan Zhang ^1^, Jingmin Yan ^1^, Bin Tang ^1^, Xiaoyi He ^1^, Jianping Xie ^3^, Xuhu Mao ^1^, Qian Li ^1*^

^1^Department of Clinical Microbiology and Immunology, College of Pharmacy and Medical Laboratory, Army Medical University (Third Military Medical University), Chongqing, 400038, China. ^2^Department of Respiratory, General Hospital of Center Theater Command, Wuhan, 400070, China. ^3^Institute of Modern Biopharmaceuticals, State Key Laboratory Breeding Base of Eco-Environment and Bio-Resource of the Three Gorges Area, Key Laboratory of Eco-environments in Three Gorges Reservoir Region, Ministry of Education, School of Life Sciences, Southwest University, Beibei, Chongqing, 400715, China. ^4^Department of General Medicine, Southwest Hospital, Army Medical University (Third Military Medical University), Chongqing, 400038, China.

^†^These authors contributed equally to this work.

**Supplemental data**

**Figures**


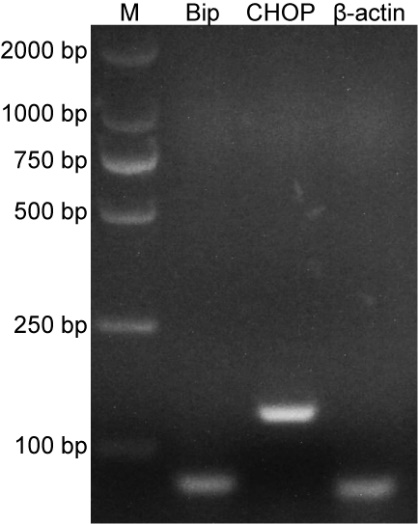


**Figure S1.** The confirmation of expected amplicon size of the primer pairs. The primer pairs were tested before qRT-PCR, using cDNA by standard PCR reaction with SimpliAmp Thermal Cycler (Thermofisher). The amplicons sizes were verified in 2% agarose gel electrophoresis.


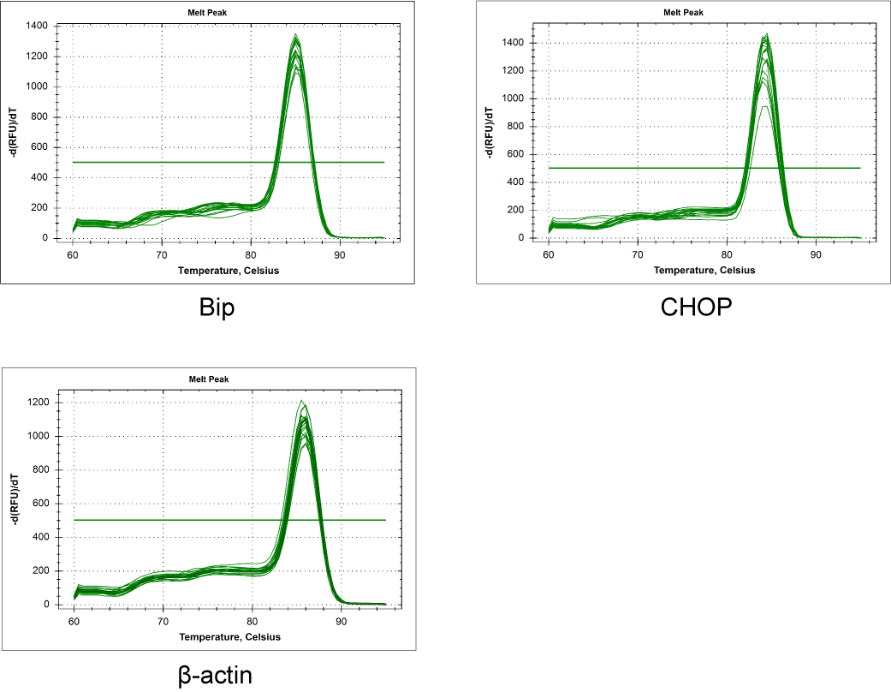


**Figure S2.** Melt curves of the Bip, CHOP and actin genes.


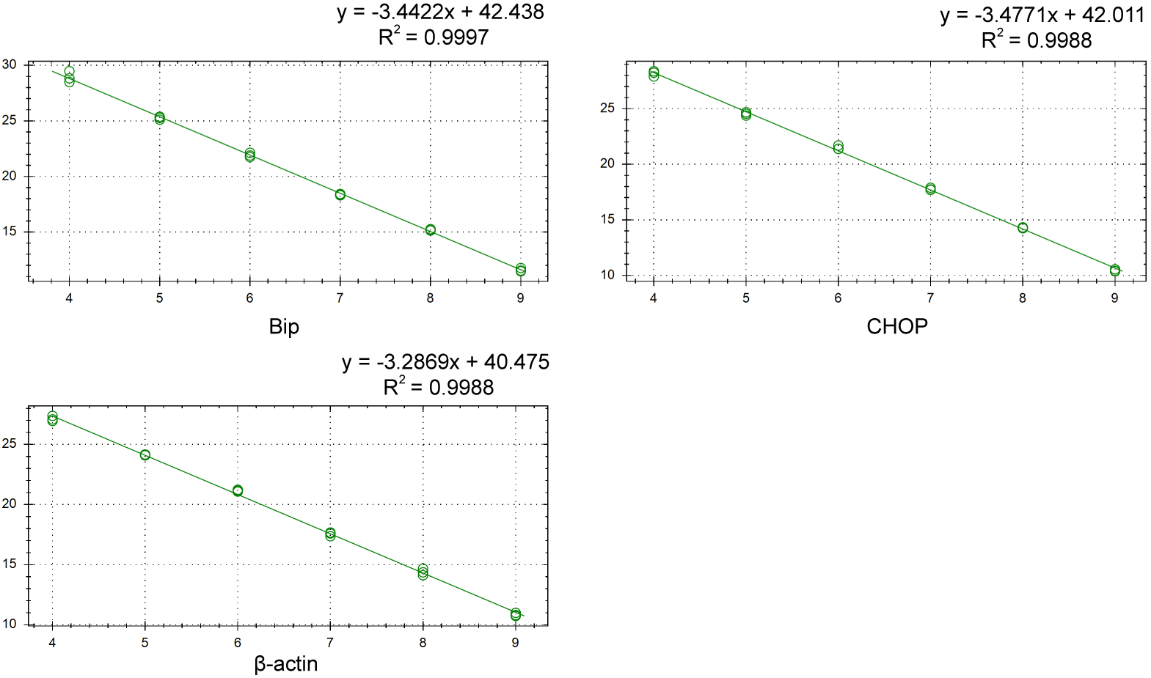


**Figure S3.** Amplification efficiencies of the qRT-PCR primers designed in the study.


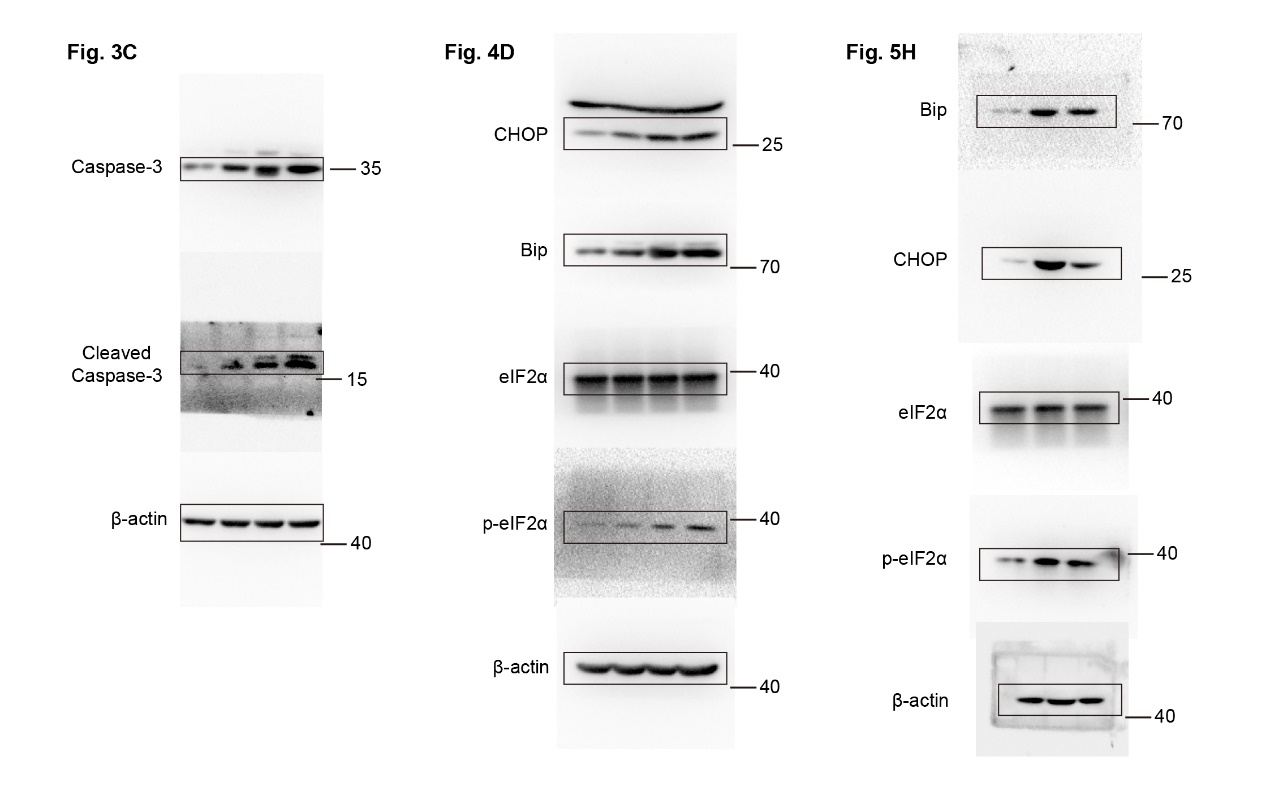


**Figure S4.** The primary images for the cropped blots in Figure 3C, 4D and 5H.


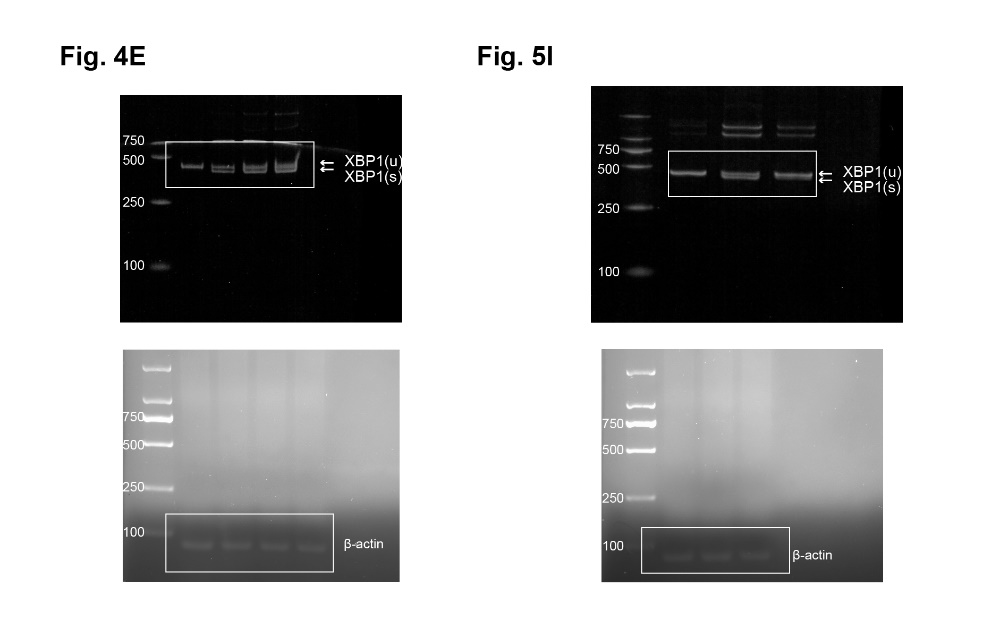


**Figure S5.** The primary images for the cropped gels in Figure 4E and 5I.


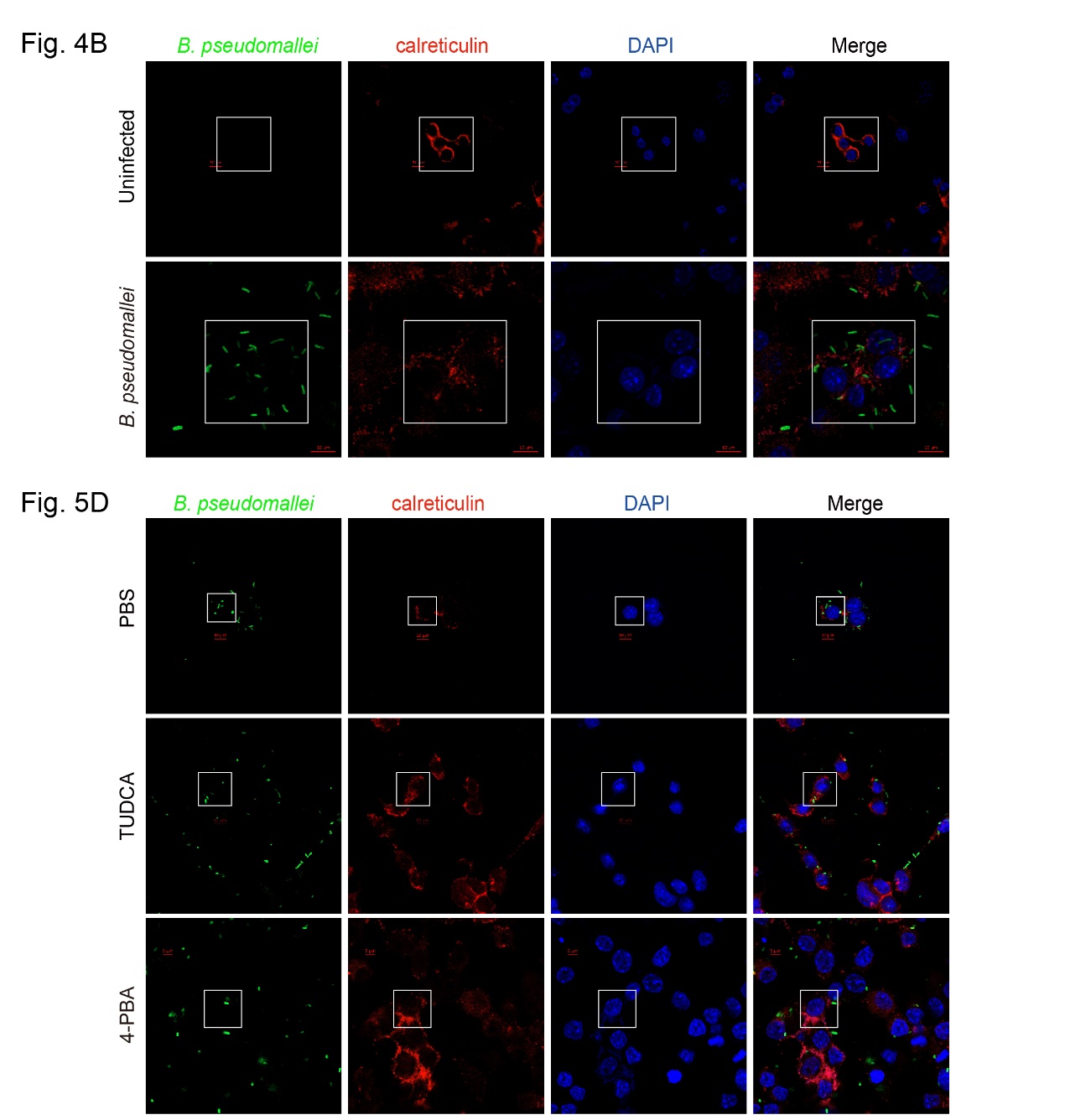


**Figure S6.** The primary confocal data for the cropped images in Figure 4B and 5D.
